# The Effect of Water Stress on Bioactive Compounds in Australian-Grown Black Sesame

**DOI:** 10.3390/plants13060793

**Published:** 2024-03-11

**Authors:** Beatriz E. Hoyos, Joel B. Johnson, Janice S. Mani, Ryan J. Batley, Tieneke Trotter, Surya P. Bhattarai, Mani Naiker

**Affiliations:** 1 School of Health, Medical & Applied Sciences, CQUniversity Australia, Bruce Hwy, Rockhampton, QLD 4702, Australia; b.hoyosortiz@cqumail.com (B.E.H.); janice.mani@cqumail.com (J.S.M.); ryan.batley@cqumail.com (R.J.B.); t.trotter@cqu.edu.au (T.T.); s.bhattarai@cqu.edu.au (S.P.B.); 2 Institute for Future Farming Systems, CQUniversity Australia, Bundaberg Campus, Bundaberg Central, QLD 4670, Australia; 3 Centre for Nutrition and Food Sciences, Queensland Alliance for Agriculture and Food Innovation (QAAFI), The University of Queensland, Brisbane, QLD 4067, Australia

**Keywords:** antioxidant activity, cupric ion-reducing antioxidant capacity, fatty acids, ferric-reducing antioxidant power, lignans, phytochemicals, sesame seeds, total phenolics

## Abstract

Sesame is an emerging crop of interest in Australia and has attracted widespread interest due to the health-benefitting properties of its bioactive compounds, including fatty acids, lignans, and polyphenols. This study aimed to investigate the impact of drought stress on these bioactive compounds, using eleven cultivars of black sesame seeds grown in Australia. Specific varieties responded positively to water deficit (WD) conditions, showing increased levels of TPC, FRAP, CUPRAC, and lignans. Varieties 1, 4, 7, and 12 showed significantly increased FRAP values ranging from 158.02 ± 10.43 to 195.22 ± 9.63 mg TE/100 g DW in the WD treatment compared to the well-watered (WW) treatment, whereas varieties 7, 10, 12, 13, and 18 demonstrated the highest CUPRAC values of all varieties (2584.86 ± 99.68–2969.56 ± 159.72 mg TE/100 g) across both WW and WD conditions, with no significant variations between irrigation regimes. Moreover, lignan contents (sesamin and sesamolin) were higher in varieties 1, 2, 5, and 8 grown in WD conditions. Compared to the optimal unsaturated to saturated fatty acid ratio (Σ UFA/Σ SFA ratio) of 0.45, all sesame genotypes showed superior ratios (ranging between 1.86 and 2.34). Moreover, the ω-6/ω-3 PUFA ratio varied from 33.7–65.5, with lower ratios in varieties 2, 4, 5, 8, and 18 under WD conditions. The high levels of phenolic compounds and healthy fats suggest the potential of black sesame to be incorporated into diets as a functional food. Furthermore, the enhanced phytochemistry of these cultivars in WD conditions is promising for widespread adoption. However, larger trial studies to confirm these findings across different geographic locations and seasons are warranted.

## 1. Introduction

*Sesamum indicum* Linn, belonging to the Pedaliaceae family, is a traditional health food and an annual oilseed crop of unknown origin [1,2]. It is commonly known as sesame and is among several species in the Sesamum genus. *Sesamum indicum* is considered a domesticated crop [2,3], with the whole seed and products obtained from sesame seed used for human consumption. The products derived from the seed initially involve pressing of the seed, resulting in three well-known by-products: the oilseed extract, sesame cake, and sesame sludge. The by-products have been extensively studied for numerous applications [4,5,6,7]. Sesame seeds are commonly used in Asian countries in bakery products for garnishing handcrafted bread, sticks, rolls, biscuits, buns, and crackers, and in international foods, such as the well-known “Tahini”, which is a staple in Mediterranean diets, and “halvah”, a Middle Eastern confection made by grinding sesame seeds together with other minor products [8]. Sesame has also been used as a snack food by adding honey and other minor sugar sources and is a major ingredient in hummus, a dip product resulting from chickpea flour and sesame paste [9]. Sesame oil, on the other hand, attracts significantly value for its health benefits [2,10], such as the prevention of obesity [11,12], coronary heart diseases [13,14,15], colon cancer [11,16,17], and gastrointestinal disorders [18,19] and reduction of the risk of developing diabetes [17,18,20]. Consequently, the inclusion of sesame in human diets may provide significant health benefits.

Sesame seeds have been imported to Australia since 1966 [21], and according to recent market analysis, 6740 tonnes of sesame were imported in 2016 [22]. Moreover, the global market value was around USD 6.5 billion in 2018, and the current trend forecasts future growth in the Australian demand to 9800 tonnes by 2025, reaching USD 17.77 billion by 2025 [22,23]. The Australian sesame industry shows promising growth potential, with a current focus on expanding cultivation across different territories [24]. Various field trials for commercial-scale sesame production are currently progressing in Queensland, Western Australia, Northern Territory, and New South Wales based on selecting optimum varieties with increased heat tolerance, enhanced yield, high oil content, and other quality parameters, targeting knowledge gaps in sowing techniques and weed management. Thus, there is an increased interest in commercializing and expanding this promising oilseed crop on the Australian market.

Both in Australia and globally, sesame has been extensively marketed due to its high nutritional value, which is mainly attributed to its bioactive constituents [4]. These compounds are present in the sesame seed [25,26], sesame oil [27,28,29], and their by-products. Further value-adding applications have emerged for the sesame by-product known as “sesame cake” or “press cake” [7]. It has been used in the development of biofuels and bio-packaging, as well as in the design and improvement of sesame breeding programs [4,7,30], and has been included as an ingredient in energy drinks and alcoholic beverages [31]. Hence, the large applicability of this valuable commodity explains the significance of increased understanding and expansion as an industrial crop.

The main feature of the oil extracted from sesame is its proven high level of resistance to oxidation when compared to oilseed crops such as corn, soybean, sunflower, peanut, and other popular plant oilseeds [27,32]. The antioxidative factor of sesame-derived oil has been attributed to the presence of phenolics, lignans, and other naturally occurring minerals and vitamins [4,30,32]. Among several health-promoting effects, polyphenols naturally present in sesame seed have demonstrated great antioxidant properties in preventing and/or treating diseases [31,33,34]. In addition, lignans of sesame—which include sesamin, sesamolin, pinoresinol, sesamol, sesaminol, and sesamolinol—have also played an important role in biological systems due to their strong antioxidant activities [12,35,36].

The estimation of the levels of the key bioactive compounds unique to sesame, particularly lignans, explains the relevance of investment in and expansion of this promising oilseed crop in the Australian market. Although sesame seeds and oilseed are used widely in Australia, the natural antioxidant levels and their bioactive potential in Australian-grown sesame cultivars have been scarcely reported. Therefore, this study aims to quantify the levels of total antioxidant capacity (TAC), total phenols (TP), total anthocyanin content, and individual sesame lignans (sesamin and sesamolin) in Australian-grown sesame. To our knowledge, there is little investigation which reports the total content of key lignans in each of the sesame derivatives, including sesame seeds, oil, cake, and sludge. Consequently, this investigation also aims to quantify the lignans in sesame seeds and their derivatives between different sesame varieties and irrigation conditions in the northern Australian region. This will enable the identification of optimal cultivars for the further development of this valuable crop.

## 2. Results and Discussion

### 2.1. Moisture Content

**Figure 1:**
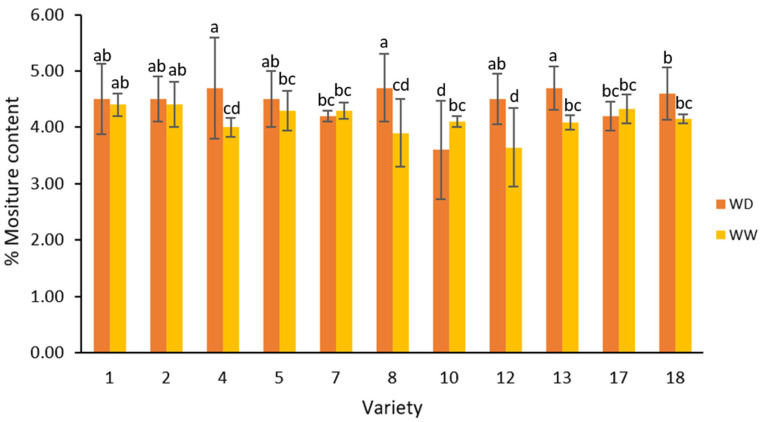
Average moisture content percentage of field-grown sesame under two irrigation treatments; well-watered (WW) and water deficit (WD). Note: ANOVA testing was based on moisture content data (*p* < 0.05). Varieties with the same lowercase letter on the bars are not significantly different, *p* < 0.05. Abbreviations: WD, water-deficient irrigation treatment; WW, well-watered irrigation treatment.

The moisture content determined for each ground sesame is presented in Figure 1. There was a significant difference noted in the moisture contents of the eleven sesame varieties (two-way ANOVA; F = 3.87, *p* < 0.05); however, there was no significant difference in the percentage of moisture content between the water irrigation treatments (two-way ANOVA; F = 3.87, *p* > 0.05), except for varieties 4, 8, 10, 12, and 13. The average moisture content ranged from 3.60–4.70% for the water deficit (WD) treatment and 3.64–4.40% for the well-watered (WW) treatment (Figure 1).

Observations from this study indicate that varieties 4, 8, and 12 had larger differences in the average moisture values amongst water irrigation treatments (Figure 1). Most of the sesame samples had higher average moisture content in the WD treatment than in the WW treatment, with only variety 10 recording a greater moisture content than its WW counterpart (Figure 1). This may indicate a certain degree of water-deficit resistance in these cultivars. Additionally, varieties 1, 2, 5, 7, and 17 had no significant differences. Thus, the results from this study may suggest that higher levels of moisture content in the seed may be due to an adaptative response of the plant to water stress [37].

The level of variation in moisture content between the sesame samples was similar to international studies on this crop [10,38,39,40,41]. For example, they were comparable to the values reported in four sesame varieties from Turkey (4.16–4.62%) [41] and somewhat comparable to commercially sourced white sesame in India (3.26–4.18%) [39]. Rostami et al. [40] reported a higher range in Iranian sesame genotypes (4.5–6.5%) than the values reported in this study. These differences may be attributed to various factors such as genetic differences, environmental conditions during cultivation, harvesting, processing methods, and storage practices [12,22,42,43].

Higher levels of moisture content in the seeds have been associated with a reduction in the oil content of the final product [40], which ultimately leads to more susceptibility to mould growth and microbial development [44]. These factors can adversely affect product quality and safety.

### 2.2. Phytochemical Profile

#### 2.2.1. Total Phenolic Content (TPC)

**Figure 2:**
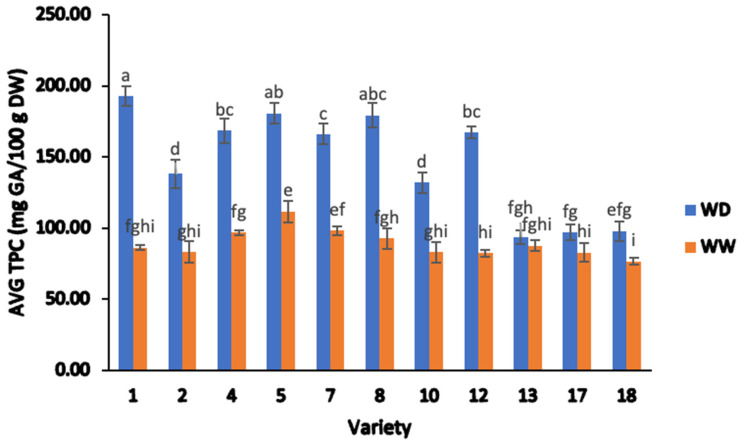
Average total phenolic content (TPC) of sesame seed from different varieties, separated by water deficient and well-watered treatments. Note: ANOVA testing was based on TPC data (*p* < 0.05). Tukey’s HSD average separation was used to distinguish between different varieties and two different irrigation treatments. Varieties with the same lowercase letter on the bars are not significantly different, *p* < 0.05. Abbreviations: WD, water-deficient irrigation treatment; WW, well-watered irrigation treatment. Analyses were performed in duplicate for each sample.

The average TPC determined for all the field-grown samples is presented in Figure 2. The phenolic content for the field-grown sesame determined with the Folin–Ciocalteu (FC) assay ranged from 76.65 mg of gallic acid equivalents (GAE) per 100 g to 192.85 mg of GAE/100 g on a dry-weight (DW) basis across both irrigation treatments. A significant difference was observed for the TPC between sesame varieties (two-way ANOVA; F = 120.60, *p* < 0.05). The average TPC values under WD and WW treatments were 146.68 ± 38.87 and 89.27 ± 9.96 mg GAE/100 g DW, respectively. Moreover, there was a significant difference in the TPC contents due to the WD treatment (two-way ANOVA; F = 2180.40, *p* < 0.05). The average TPC of each sesame variety ranged from 93.53 to 192.85 mg GAE/100 g DW in the WD treatment, while sesame grown in the WW treatment ranged from 76.65 to 111.72 mg GAE/100 g DW. It was also noted that there was a significant impact on each of the varieties subjected to the two water treatment conditions (two-way ANOVA; F = 255.10, *p* < 0.05) (Figure 2).

The TPC content was significantly higher in the WD treatment for most of the sesame varieties when compared to the WW treatment. For example, the phenolic content was approximately two-fold higher in varieties 1 (193.85 ± 6.83 mg GAE/100 g DW) and 12 (167.42 ± 4.20 mg GAE/100 g DW) in the WD treatment than in the WW treatment (86.20 ± 1.70 and 82.39 ± 2.21 mg GAE/100 g DW, respectively). Across the remaining nine varieties, WD treatment provided a 48.25%, 42.42%, 40.81%, and 39.71% increase in TPC for varieties 8, 4, 7, and 2, while varieties 18, 17, and 13 only showed a 21.82%, 14.71%, and 6.33% increase, respectively. This is indicative of a wide range of genetic differences in both the total phenolic content of the varieties and their physiological responses to water stress [45,46,47].

The current results were higher than the results reported in white sesame by Elleuch et al. [10], the value of which was 87.8 mg GAE/100 g DW in Tunisia. Moreover, the TPC results obtained in the current study were significantly lower than previous values reported in China (370.5–786.8 mg of GAE/100 g DW) [33] and Ethiopia (370.5–786.8 mg of GAE/100 g DW) [48]. Another previous investigation in India [49] obtained comparable TPC values (126.8–154.3 mg of GAE/100 g DW) to the current investigation. In addition, comparable values were reported in a previous investigation using ten dried and milled sesame genotypes (including black, brown, light brown, and white colors) of different origins worldwide and two different irrigation treatments [46] in Iran. Their TPC values ranged from 76.0–262.0 mg GAE /100 g DW and 86.7–334.0 mg GAE /100 g DW in the assessment of full irrigation (60% irrigation in the soil) and 90% in deficient irrigation, respectively. In general, previous studies have found TPC ranging from 76.06 to 2569.0 mg of GAE/100 g DW [10,33,48,49]. It should be noted that the whole seeds were analyzed in the present study, while some previous researchers have investigated dehulled seeds. In general, lower TPC values are found in the whole seed extracts compared to hull extracts [50]. Nevertheless, the relatively high TPC values found here for whole black sesame seeds suggests that they may provide beneficial health effects.

The statistically significant variation within the WW and WD groups also indicated that the genotypes of sesame reacted differently to water deficiency in terms of TPC. Similar observations have been made for other crops such as cumin seeds and rapeseed, in which water deficiency also affected the concentrations of these secondary metabolites [46,51]. Okello [52], Sánchez-Rodríguez [53], and Bettaieb [54] noted extensive variation in TPC (76.0–2569.0 mg GAE/100 g DW) amongst plants in the Solanaceae family such as huckleberry (*Solanium scabrum* Mill.) and tomatoes, and in cumin (*Cuminum cyminum* L.) in the Apiaceae family, varying widely depending on different factors such as genetics, environmental conditions during cultivation, harvesting practices, processing methods, and storage practices [45,46,47]. Based on the discussed information, it can be postulated that the accumulation of phenolic compounds in sesame seeds could be due to the plant’s response to water stress. This can be based on previous studies that have shown that plants often produce more phenolic compounds in response to stress factors such as environmental stress, disease, or insect attack [55,56,57]. This increase in secondary metabolites serves as an adaptive strategy that has evolved over time to help plants cope with a wide range of abiotic stresses. This adaptive behaviour often involves the activation of specific metabolic pathways. The phenylpropanoid biosynthetic pathway is likely the pathway associated with the secondary metabolism behind the stimulated biosynthesis of phenolic compounds induced by abiotic stresses, such as water deficit [58]. Water deficit regulates many key genes encoding the main enzymes of the phenylpropanoid pathway, which results in stimulated biosynthesis of phenolic compounds [59]. However, it is important to note that there may be other factors that can contribute to the variation in phenolic content, such as the genetic diversity of the sesame varieties studied [10,33,49,60].

#### 2.2.2. Ferric-Reducing Antioxidant Power (FRAP)

**Figure 3:**
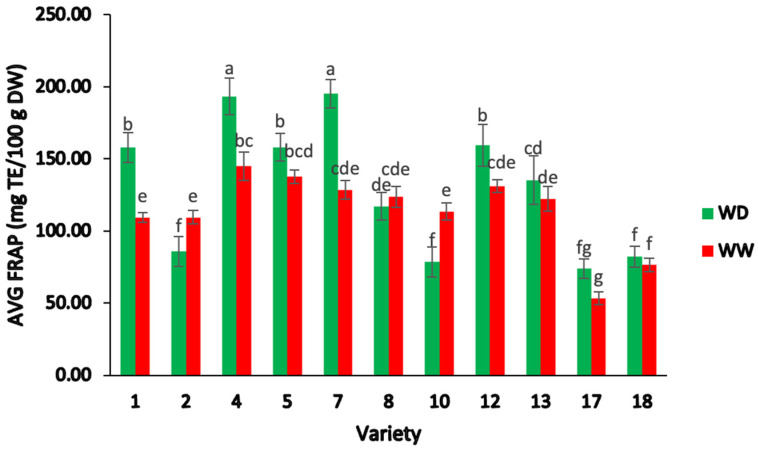
Average ferric-reducing antioxidant power (FRAP) of different varieties of sesame seed, separated by water deficient and well-watered treatments. Note: ANOVA testing (*p* < 0.05) was based on FRAP data. Tukey’s HSD average separation was used to distinguish between different varieties and two different irrigation treatments. Varieties with the same lowercase letter on the bars are not significantly different, *p* < 0.05. Abbreviations: TE, Trolox equivalent; DW, dry weight; WD, water-deficient irrigation treatment; WW, well-watered irrigation treatment. Analyses were performed in duplicate for each sample.

The average FRAP content (Figure 3) for the field trial samples varied significantly, ranging from 53.53–195.22 mg of Trolox equivalents (TE) per 100 g DW, including both irrigation treatments (two-way ANOVA; F = 123.60, *p* < 0.05). 

There was a significant difference amongst the average FRAP values for the irrigation treatments, which ranged from 74.04–195.22 mg TE/100 g DW under WD treatment and 53.53–145.00 mg TE/100 g DW under WW treatment (two-way ANOVA; F = 69.51, *p* < 0.05). In addition, FRAP levels varied significantly between varieties and water treatments (two-way ANOVA; F = 29.52, *p* < 0.05) (Figure 3). These results indicate that the antioxidant activity of the eleven sesame genotypes was influenced by water deficiency, but the extent of the response to this stress varied greatly depending on the variety. Varieties 7, 1, and 4 showed 34.10%, 30.67%, and 24.99% higher FRAP values under WD conditions than in the WW treatment. In contrast, the FRAP of varieties 10 and 2 were 30.64% and 21.51% greater under the WW treatment. In addition, varieties 5, 8, 12, 13, 17, and 18 did not differ significantly between the two water treatments.

The FRAP values observed in the sesame varieties analyzed in this study were generally comparable to previous reports concerning a range of other crops [10,46,48,49,61,62,63,64,65]. The FRAP values reported by Mercado et al. [66] represented a wider range (0.75–425 mg TE/100 g DW), which may be due to genetic variation in the eight Iranian sesame genotypes used in this study or different extraction parameters. The differences found amongst the FRAP values obtained in the current study and the values reported globally could be also attributed to the type, diversity, and concentration of different polyphenolic compounds making up the different extracts [67]. It is believed that combinations of various minor constituents present in sesame seeds may work synergistically to enhance the antioxidant activity. This has been supported by findings from previous studies [67,68,69,70,71,72,73].

Our findings on the interaction between water stress and genotypes in this study align with previous efforts conducted by Kim et al. [74], who suggested that the activity of potential antioxidant compounds in sesame is affected by water deficiency stress; however, the response to water deficit differs depending on the sesame cultivar. In line with other studies [47,74], the antioxidant activity measured by FRAP was generally dependent on the water stress level and sesame genotype. In this study, the genotypes with higher FRAP values in the WW treatment can be categorized as sensitive to water stress, as described in other studies [47,74]. Previous efforts have shown that plants generally produce more phenolic, lignan, tocopherol, and sterol contents when subjected to environmental stress, disease, or insect attack [55,56,57]. Thus, the differences in antioxidant activity observed in the different sesame samples studied may be attributed to physiological changes experienced by the plants as a defence mechanism by enhancing the bioactive content. However, it is important to consider that other factors such as the diversity of sesame varieties studied may also contribute to the variation in antioxidant capacity, as postulated by previous investigations [10,33,49,60].

#### 2.2.3. Cupric Ion-Reducing Antioxidant Capacity (CUPRAC)

**Figure 4:**
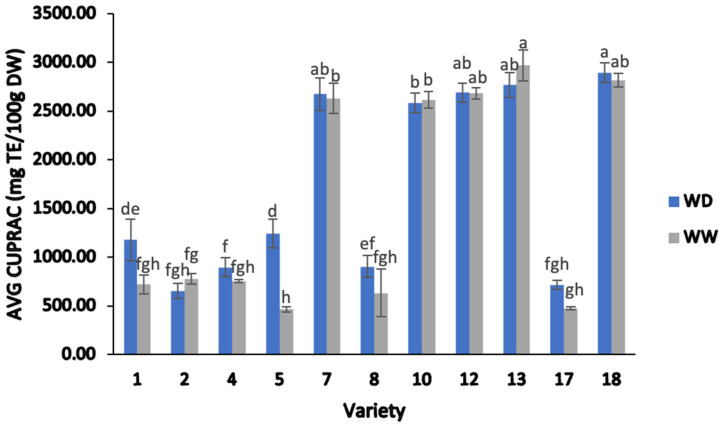
Average cupric ion-reducing antioxidant capacity (CUPRAC) values of sesame seed from different varieties, separated by water-deficient and well-watered treatments. Note: ANOVA testing (*p* < 0.05) was based on CUPRAC data. Tukey’s HSD average separation was used to distinguish between different varieties and two different irrigation treatments. Varieties with the same lowercase letter on the bars are not significantly different, *p* < 0.05. Abbreviations: WD, water-deficient irrigation treatment; WW, well-watered irrigation treatment. Analyses were performed in duplicate for each sample.

The CUPRAC assay was performed as an alternative method of investigating the effect of water deficit stress on the antioxidant capacity of the various sesame cultivars. The average CUPRAC values determined for the eleven field trial varieties are presented in Figure 4. 

There was a significant difference noted in the antioxidant activity results obtained by the CUPRAC method in the eleven sesame varieties, ranging from 465.99–2969.56 mg of Trolox equivalents (TE) per 100 g DW (two-way ANOVA; F = 707.84, *p* < 0.05). In addition, there was a significant impact noted amongst the average CUPRAC values for the irrigation treatments, which ranged from 655.81–2896.05 mg TE/100 g DW for WD treatment, while these values ranged from 465.99–2969.56 mg TE/100 g DW for WW treatment (two-way ANOVA; F = 155.60; *p* < 0.05). These observed results suggest that the antioxidant activity of the eleven sesame genotypes was influenced by the irrigation treatment and, furthermore, that responses to such treatment differed depending on the sesame cultivar. For example, varieties 5 and 1 showed 62.47% and 38.78% higher CUPRAC values under WD treatment compared to the WW treatment. Although varieties 13 and 10 did show slightly higher CUPRAC values under WW treatment, these, along with all other varieties, did not show any statistically significant difference between irrigation treatments.

The evaluation of antioxidant activities using the FRAP and CUPRAC methods revealed notable variability across all sesame samples. The CUPRAC method exhibited generally higher levels of antioxidant activity than the FRAP method. While sesame samples from varieties 1, 4, 7, and 12 subjected to WD treatment showed greater antioxidant activity than those with WW treatment, a few exceptions (varieties 2 and 10) displayed higher antioxidant values in the WW treatment when tested using the FRAP assay. This divergence may suggest a potential genotype-dependent response. Meanwhile, varieties 1 and 5 demonstrated higher antioxidant activity (around twofold and threefold) in the WD treatment than in the WW treatment, as determined by the CUPRAC method. Due to the lack of studies in the literature reporting CUPRAC data, a comparison of CUPRAC levels in sesame seeds was not able to be performed. Taken together, the results of this study and the potential explanations given for other species highlight the need for further investigation of individual phenolics and their potential contribution to the overall antioxidant activity. 

### 2.3. Determination of Lignans in Sesame Seeds Using HPLC Analysis

#### 2.3.1. Sesamin

**Figure 5:**
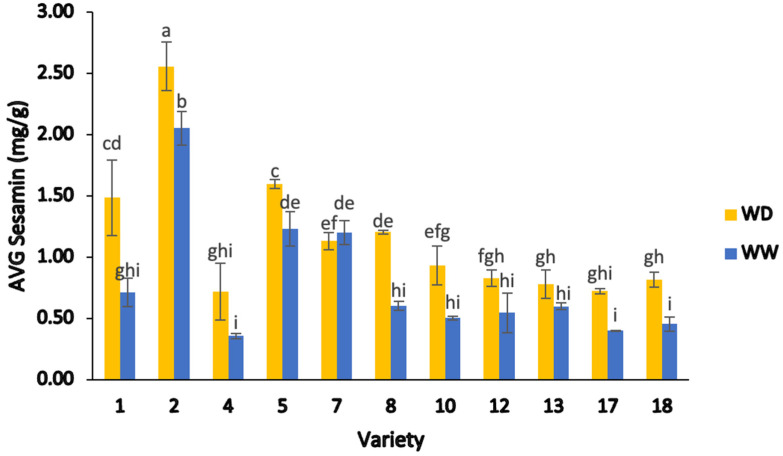
Average sesamin content (mg/g) of whole sesame seed from different varieties, separated by water deficient and well-watered treatments. Note: ANOVA testing (*p* < 0.05) was based on HPLC data. Tukey’s HSD average separation was used to distinguish between different varieties and two different irrigation treatments. Varieties with the same lowercase letter on the bars are not significantly different, *p* < 0.05. Abbreviations: WD, water-deficient irrigation treatment; WW, well-watered irrigation treatment. Analyses were performed in duplicate for each sample.

The average sesamin content in the whole black sesame seed extracts (Figure 5) varied significantly amongst the eleven lines, ranging from 0.36–2.56 mg/g, with significant differences between varieties (two-way ANOVA; F = 174.86, *p* < 0.05). Moreover, there was a significant difference between seeds from the WD irrigation treatment (0.60–2.56 mg/g) and the WW treatment (0.36–2.05 mg/g; two-way ANOVA; F = 189.30, *p* < 0.05). In addition, there was a significant interaction between varieties and irrigation treatments (two-way ANOVA; F = 22.24, *p* < 0.05) (Figure 5). Among the eleven genotypes analyzed, the highest average sesamin content was observed for variety 2 (2.56 mg/g) under WD treatment, followed by variety 2 under WW treatment (2.05 mg/g) and variety 5 under WD treatment (1.60 mg/g) (Figure 5). The lowest average sesamin value was observed in variety 4 under WW treatment (0.36 mg/g).

Water stress influenced sesamin content in some varieties, specifically increasing the average values in the WD treatment [46]. For example, varieties 1, 4, 18, and 17 were 52.17%, 50.47%, 44.51%, and 44.36% higher in average sesamin content in the WD treatment than in the WW treatment (Figure 5). On the other hand, variety 7 was unique in showing a greater sesamin content in the WW treatment than the WD treatment, although the difference between treatments was not statistically significant (Figure 5).

These observed results indicate that water deficit treatment appeared to affect the sesamin content in sesame samples, as evidenced by a higher sesamin content in most of the sesame samples subjected to WD treatment, with the exception of varieties 4, 7, 13, and 17. More specifically, in varieties subjected to WD conditions, 45% of the genotypes (varieties 1, 2, 5, 7, 8) exhibited sesamin contents of greater than 1.0 mg/g, whilst 55% of the samples (varieties 4, 10, 12, 13, 17, and 18) had from 0.7–1.0 mg/g. In contrast, under WW treatment, only 27% of the samples (varieties 2, 5, and 7) exhibited average sesamin values greater than 1.0 mg/g, whereas 64% of genotypes (varieties 1, 8, 10, 12, 13, 17, and 18) had average values below 1.0 mg/g. As can be seen in Figure 5, the response to the irrigation treatment was different depending on the sesame cultivars. For example, varieties 8, 10, and 12 were 50.02%, 46.14%, and 34% higher in the average sesamin values for the WD treatment than the same varieties in the WW treatment. The average sesamin values in varieties 13, 5, and 2 were greater (23.54%, 23.01%, and 19.74%, respectively) for the WD group than for the WW group. 

The average sesamin content in this study was comparable to values reported in a previous Korean study [74], ranging from 0.97–3.76 mg/g under WD treatment and from 0.31–4.12 mg/g under WW treatment in eighteen sesame genotypes investigated. Other studies attributed the variation in the average sesamin ranges to different seedcoat colors and irrigation regimes [46]. For example, a study on ten Iranian genotypes found that the average sesamin values were higher in dark-colored sesame seeds subjected to WW treatment (1.67–3.05 mg/g; average of 3.45 mg/g) than those same genotypes subjected to WD treatment (0.49–2.47 mg/g; average of 2.68 mg/g).

#### 2.3.2. Sesamolin

**Figure 6:**
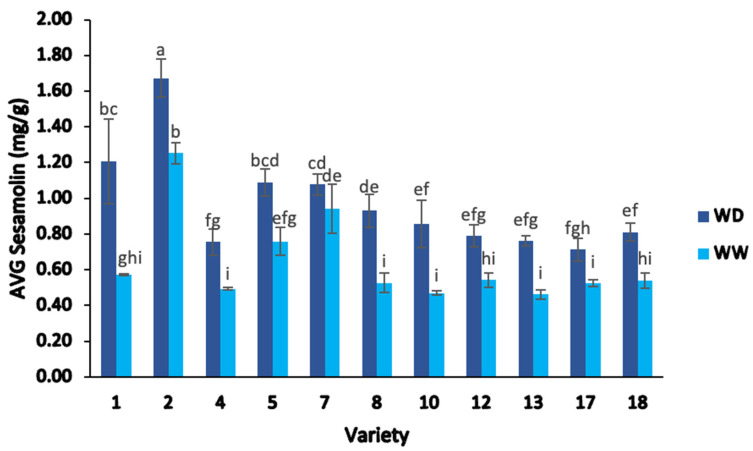
Average sesamolin content (mg/g) of sesame seed from different varieties, separated by water deficient and well-watered treatments. Note: ANOVA testing (*p* < 0.05) was based on HPLC data. Tukey’s HSD average separation was used to distinguish between different varieties and two different irrigation treatments. Varieties with the same lowercase letter on the bars are not significantly different, *p* < 0.05. Abbreviations: WD, water-deficient irrigation treatment; WW, well-watered irrigation treatment. Analyses were performed in duplicate for each sample.

The average sesamolin content (Figure 6) varied significantly amongst the eleven lines, ranging from 0.46–1.67 mg/g in the whole seeds, with significant differences between varieties and irrigation treatments (two-way ANOVA; F = 43.71, *p* < 0.05). 

There was a significant difference amongst the average sesamolin values between varieties, which ranged from 0.71–1.67 mg/g under WD treatment and 0.46–1.25 mg/g under WW treatment (two-way ANOVA; F = 429.97, *p* < 0.05). In addition, there was a significant interaction between varieties and water treatments in the sesamolin levels (two-way ANOVA; F = 116.73, *p* < 0.05), (Figure 6). 

Among the eleven genotypes analyzed, the highest average sesamolin content was observed again in variety 2 (1.67 mg/g) under WD treatment, followed by variety 2 under WW treatment (1.25 mg/g) and variety 1 under WD treatment (1.21 mg/g) (Figure 6). The lowest average sesamolin value was found in variety 13 under WW treatment (0.46 mg/g) (Figure 6).

Compared to the sesamin content, the effect of WD treatment was generally more evident in sesamolin content across almost all sesame genotypes, except in variety 7, which showed no significant differences in sesamolin content between the two irrigation treatments (WD and WW). The average sesamolin content was 33.5% higher in the WD treatment compared to the WW treatment (Figure 6). Similarly, 36% of the samples (varieties 2, 1, 5, and 7) in the WD treatment exhibited a sesamolin content higher than 1.0 mg/g, whilst only 9% of the samples (variety 2) had a sesamolin value greater than 1.0 mg/g under WW treatment.

Nevertheless, the response to water stress did differ depending on the cultivar. Most of the sesame varieties had higher average sesamolin values under the WD treatment than in the WW treatment, with prominent differences in varieties 1 (52.5%), 10 (45%), 8 (43%), 13 (39%), and 4 (35%). Only variety 7 had no significant difference in the average sesamolin content between the two irrigation treatments (WD and WW), with values of 1.08 mg/g and 0.94 mg/g, respectively.

Previous authors such as Wang et al. [75], Dossa et al. [76], and Dar et al. [60] compared the amount of lignans in differently colored sesame seeds (white, yellow, brown, and black) across a large number of accessions. These authors found that white sesame seeds had higher levels of lignans than the other sesame genotypes. Likewise Shi et al. [77], Dar et al. [60], Muthulakshmi et al. [78], and Kim et al. [74] found that black-colored seeds contained larger quantities of sesamin and sesamolin contents. Dar et al. [79] reported an average sesamolin content of 1.56–3.28 mg/g in black sesame, slightly higher than the range of 0.46–1.67 mg/g found in the present study. Again, this difference may be due to genotypic variation or different environmental conditions.

There is also limited previous research on the influence of irrigation treatments on the phytochemical composition of sesame seeds, particularly with respect to lignans. Several studies have reported a slight increase in the average sesamin content under water-deficit treatment [2,74,80]. However, ref. [76] reported no significant variation in the sesamolin content of ten African sesame genotypes under water stress.

The primary cause behind the wide variations in lignan content is likely induced during irrigation deficit conditions, in which there is an adjustment of the phenylpropanoid biosynthetic pathway [59,81]. Water deficit influences several crucial genes responsible for encoding essential enzymes in the phenylpropanoid pathway, leading to increased production of various bioactive compounds to protect the plant from unfavourable environmental conditions [25,59]. While acknowledging the necessity for additional research in this domain, this study tentatively proposes that inducing water deficit may represent a viable strategy for increasing lignan content in sesame seeds; however, a broader range of genotypes should be examined to fully understand the effects of different irrigation regimes on the phytochemical composition in sesame seeds.

### 2.4. FAME Analysis by GC–MS in Sesame Seeds

**Figure 7:**
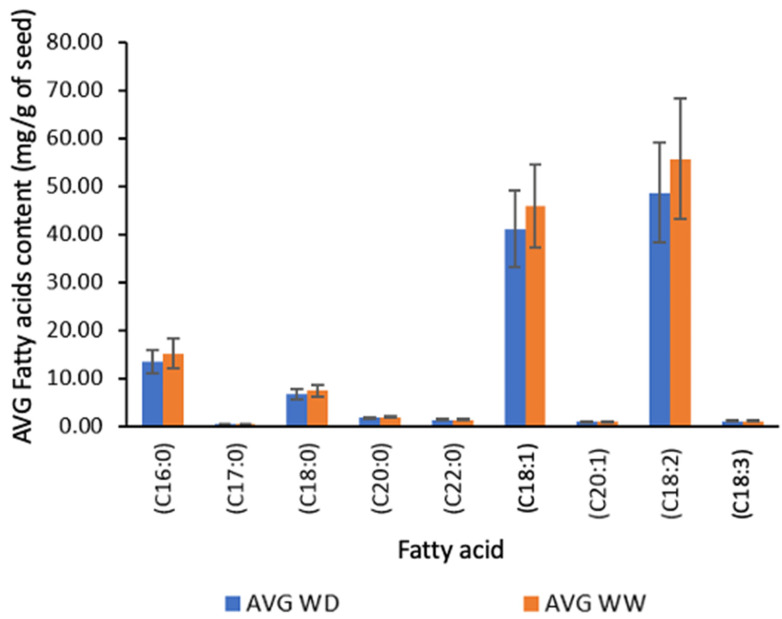
Average fatty acid content (mg/g) of sesame seeds from different varieties, categorized by water deficient and well-watered treatments. Note: ANOVA testing was based on GC–MS data (*p* < 0.05). Tukey’s HSD average separation was used to distinguish between two different irrigation treatments. Abbreviations: AVG: average; WD, water-deficient irrigation treatment; WW, well-watered irrigation treatment; C16:0, palmitic acid; C17:0, margaric acid; C18:0, stearic acid; C18:1, oleic acid; C18:2, linoleic acid; C18:3, a-linolenic acid; C20:0, arachidic acid; C22:0, behenic acid. Analyses were performed in duplicate for each sample.

Sesame seeds are recognized for their diverse fatty acid compositions, with notable constituents including linoleic acid, oleic acid, palmitic acid, and stearic acid [3,10,41,82,83,84,85,86]. Nine fatty acids were more prevalent in all the eleven ground sesame varieties under both WD and WW irrigation treatments, as indicated in Figure 7. The fatty acid profiles of the eleven black sesame varieties are categorized based on carbon saturation levels into saturated fatty acids (SFA), monounsaturated fatty acids (MUFA), and polyunsaturated fatty acids (PUFA).

The composition of these fatty acids in sesame seeds varies with the seed variety and environmental factors such as soil type, weather, and growing conditions [3,60,87,88]. The SFAs, such as palmitic acid (C16:0), margaric acid (C17:0), stearic acid (C18:0), arachidic acid (C20:0), and behenic acid (C22:0), were present in all the black sesame varieties. The MUFAs, like oleic acid (C18:1) and cis-11-eicosenoic (C20:1), were also detected across all varieties. Additionally, PUFAs such as linoleic acid (C18:2) and α-linolenic acid (C18:3; cis-9,12,15) were identified. 

The SFA profile of sesame seeds, particularly palmitic acid (C16:0) and stearic acid (C18:0), showed notable differences based on irrigation treatment. Palmitic and stearic acid levels were lower in the WD treatment (13.34 ± 2.49 and 6.56 ± 1.10 (mg/g)) compared to the WW treatment (15.11 ± 3.07 and 7.35 ± 1.25 (mg/g) of seed). Significant differences (two-way ANOVA; *p* < 0.05) in the levels of various SFAs were observed among sesame varieties under both irrigation treatments. Specifically, palmitic acid, stearic acid, arachidic acid, and behenic acid exhibited significant differences, ranging from 9.00–21.93 (F = 9.75, *p* < 0.05), 4.63–10.06 (F = 8.39, *p* < 0.05), 1.54–2.14 (F = 9.75, *p* < 0.05), and 1.30–1.43 (F = 11.00, *p* < 0.05) mg/g, respectively. However, no significant difference (two-way ANOVA; F = 1.03, *p* > 0.05) was observed in margaric acid levels under both treatments, ranging between 0.50 and 0.56 mg/g. Furthermore, the major SFA content varied significantly among sesame genotypes and irrigation treatments. For example, higher palmitic acid and stearic acid contents were observed in the WW treatment (11.44–21.93 mg/g and 5.73–10.03 mg/g, respectively) compared to the WD treatment (9.00 to 16.73 mg/g and 4.63 to 8.0.7 mg/g, respectively), indicating a greater influence of WW treatment on major SFA levels.

**Figure 8:**
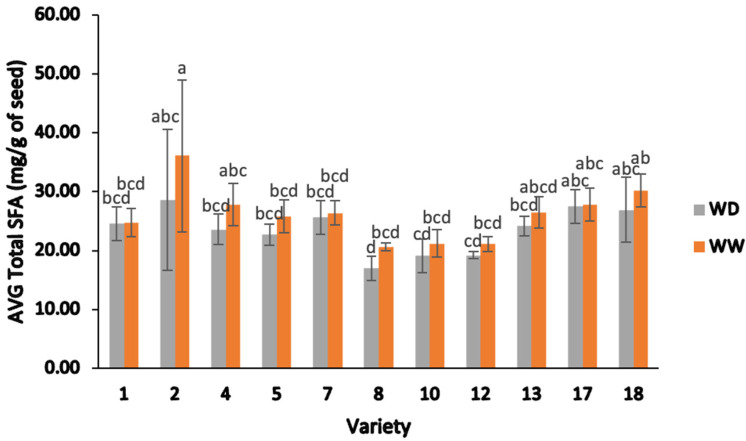
Average total saturated fatty acid (SFA) content (mg/g) of different varieties of sesame seed, separated by water-deficient and well-watered treatments. Note: ANOVA testing was based on GC–MS data (*p* < 0.05). Tukey’s HSD average separation was used to distinguish between different varieties and two different irrigation treatments. Varieties with the same lowercase letter on the bars are not significantly different, *p* < 0.05. Abbreviations: AVG: average; Total SFA, total saturated fatty acids; WD, water-deficient irrigation treatment; WW, well-watered irrigation treatment. Analyses were performed in duplicate for each sample.

**Figure 9:**
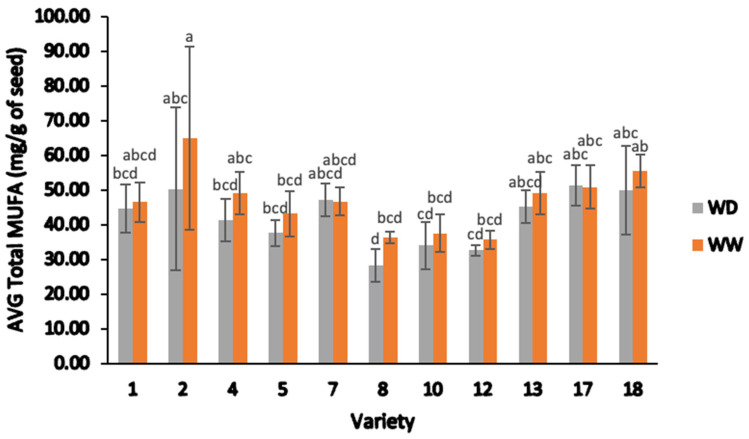
Average total monounsaturated fatty acid (MUFA) content (mg/g) of different varieties of sesame seeds, categorized by water deficient and well-watered treatments. Note: ANOVA testing was based on GC–MS data (*p* < 0.05). Tukey’s HSD average separation was used to distinguish between different varieties and two different irrigation treatments. Varieties with the same lowercase letters on the bars are not significantly different, *p* < 0.05. Abbreviations: AVG, average; Total MUFA, total monounsaturated fatty acids; WD, water-deficient irrigation treatment; WW, well-watered irrigation treatment. Analyses were performed in duplicate for each sample.

Figure 8 illustrates the total saturated fatty acids (Total SFA content) derived from each field-grown sample, categorized by varety and irrigation condition. Variety 2 had higher average total MUFAs than all other varieties for WW and had the second highest average total MUFAs after variety 17 (Figure 9). Only variety 8 had a statistical difference between WD and WW, with WW having a higher value.

The average MUFAs, specifically oleic acid (C18:1) and cis-11-eicosenoic acid (C20:1), responded differently to the irrigation treatments. Oleic acid showed a significant difference (F = 8.90, *p* < 0.05), ranging from 27.38 to 63.91 mg/g, compared to cis-11-eicosenoic acid (F = 9.60, *p* < 0.05), which ranged from 0.85 to 1.05 mg/g. Varieties and irrigation treatments significantly influenced the average MUFA levels. Oleic acid content ranged from 27.38 to 50.33 mg/g under WD treatment and from 34.78 to 63.91 mg/g under WW treatment. The average cis-11-eicosenoic acid content was slightly lower under WD treatment (0.85 to 0.97 mg/g) than under WW treatment (0.88 to 1.05 mg/g). Variety 2 exhibited the highest average oleic acid content (63.91 mg/g) under WW treatment, while variety 8 had the lowest (27.38 mg/g) under WD treatment. These findings suggest that water stress significantly affected oleic acid content in some genotypes, particularly varieties 2, 8, 4, and 5, while cis-11-Eicosenoic acid content was less influenced by water stress, as was observed in varieties 2, 12, and 5. 

**Figure 10:**
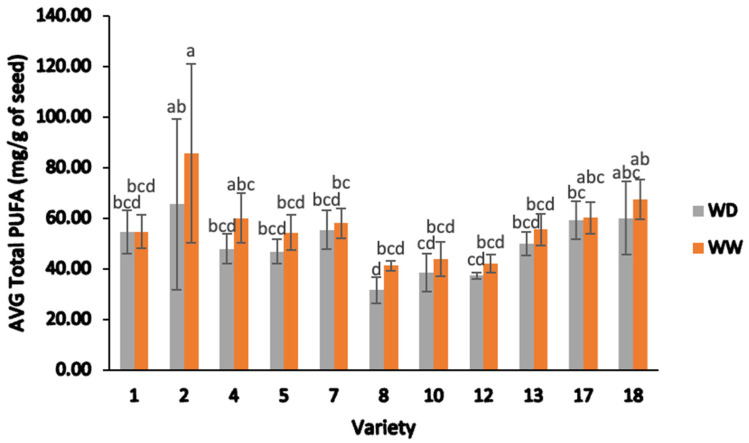
Average total polyunsaturated fatty acid (PUFA) content (mg/g) of different varieties of sesame seed, separated by water deficient and well-watered treatments. Note: ANOVA testing was based on GC–MS data (*p* < 0.05). Tukey’s HSD average separation was used to distinguish between different varieties and two different irrigation treatments. Varieties with the same lowercase letters on the bars are not significantly different, *p* < 0.05. Abbreviations: AVG: average; Total PUFA, total polyunsaturated fatty acids; WD, water-deficient irrigation treatment; WW, well-watered irrigation treatment. Analyses were performed in duplicate for each sample.

Figure 10 displays the total polyunsaturated fatty acid (Total PUFA) content in field-grown samples, categorized by variety and irrigation. Generally, most genotypes under WD treatment showed slightly lower total PUFA levels compared to WW treatment. Varieties 1, 5, 7, 10, 13, and 17 exhibited consistent total PUFA contents across both treatments (Figure 10).

The average linoleic acid content differed significantly among the eleven lines (two-way ANOVA; F = 10.04, *p* < 0.05), ranging from 48.67 to 55.6 mg/g. There were notable differences between varieties and irrigation treatments (two-way ANOVA; F = 10.46, *p* < 0.05). Under WD treatment, linoleic acid ranged from 30.69 to 64.35 mg/g, lower than under the WW treatment (40.35 to 84.46 mg/g). However, the average α-linolenic acid content showed significant differences between irrigation treatments (two-way ANOVA; F = 1.75, *p* > 0.05), averaging 1.04 ± 0.09 and 1.06 ± 0.10 mg/g.

Among the eleven genotypes, variety 2 exhibited the highest average linoleic acid content (84.46 mg/g) under WW treatment, followed by variety 18 (66.22 mg/g) under WW treatment and variety 2 under WD treatment (64.35 mg/g). The lowest average linoleic acid value was found in variety 8 under WD treatment (30.69 mg/g). Meanwhile, average α-linolenic acid values were greater in the WW treatment for varieties 2 (7%), 4 (4.5%), 8 (4%), and 5 (3%) compared to the same varieties in the WD treatment. However, varieties 1 and 17 showed a marginal increase of around 2% in their average α-linolenic acid values under WD treatment compared to the WW treatment.

Based on the results, different irrigation regimes significantly influenced the fatty acid composition of the eleven black sesame varieties. Some varieties showed better performance in maintaining higher levels of unsaturated fatty acids and lower levels of saturated fatty acids under water-deficient conditions. For instance, variety 8 exhibited the lowest average saturated fatty acids under WD treatment, while varieties 13, 5, and 4 showed moderate ranges of unsaturated fatty acids and the second lowest average of saturated fatty acids under the same treatment. Conversely, varieties 2 and 18 had the highest average linoleic acid contents under WW treatment, indicating potential for cultivation under water-deficient conditions. However, further research is needed to fully assess their cultivation potential, considering factors like genetics, growing conditions, and plant architecture [60,75,76,89].

Previous studies [2,35,46,74,77,79,80,89] have shown that the concentration of oleic acid and linoleic acid in sesame seeds is affected by various factors, including water stress, climate, storage, and growing conditions. For instance, a study in Korea observed differences in UFA contents among eighteen genotypes under different irrigation regimes [74]. These genotypes presented differences in oleic acid and linoleic acid contents, with a wider range under WD treatment (from 40.3–45.3 mg/g and 40.9–46.1 mg/g, respectively) compared to WW treatment (from 41.0–44.3 mg/g and 42.4–46.2 mg/g, respectively) conditions. In Iran, Khorami et al. [90] found decreased unsaturated fatty acid and increased saturated fatty acid levels in a hybrid sesame genotype under different water stress levels. They observed a reduction in oleic acid from 42.4% (WW) to 40.6% (WD) and a drop in linoleic acid from 30.2% (WW) to 29.5% (WD). However, palmitic acid remained unaffected, while stearic acid increased. In Turkey, Ozkan et al. [91] reported that as water deficit severity increased, oleic acid decreased from 44.21 to 42.18 mg/g, while linoleic acid levels varied among genotypes, indicating genotype-dependent responses to water deficiency.

This study revealed varying responses in fatty acid composition among the eleven black sesame varieties under different irrigation regimes. Genotypes maintaining higher unsaturated fatty acid and lower saturated fatty acid levels under water deficiency are of significant interest due to their potential health benefits [50]. These health benefits are supported by a Σ PUFA/Σ SFA ratio exceeding 0.45 [92,93,94] and a ratio of 5~10:1 for ω-6/ω-3 PUFA, recommended by health organizations to mitigate cardiovascular diseases and other chronic conditions [93,94]. All field-grown sesame samples achieved this ratio, ranging from 1.86 to 2.34. Additionally, the ω-6/ω-3 PUFA ratio varied from 33.7 to 65.5, with lower ratios observed in specific varieties under water deficit conditions, suggesting their potential for further investigation and cultivation.

### 2.5. Correlation Analysis of Bioactive Compounds in Sesame Seeds

**Figure 11:**
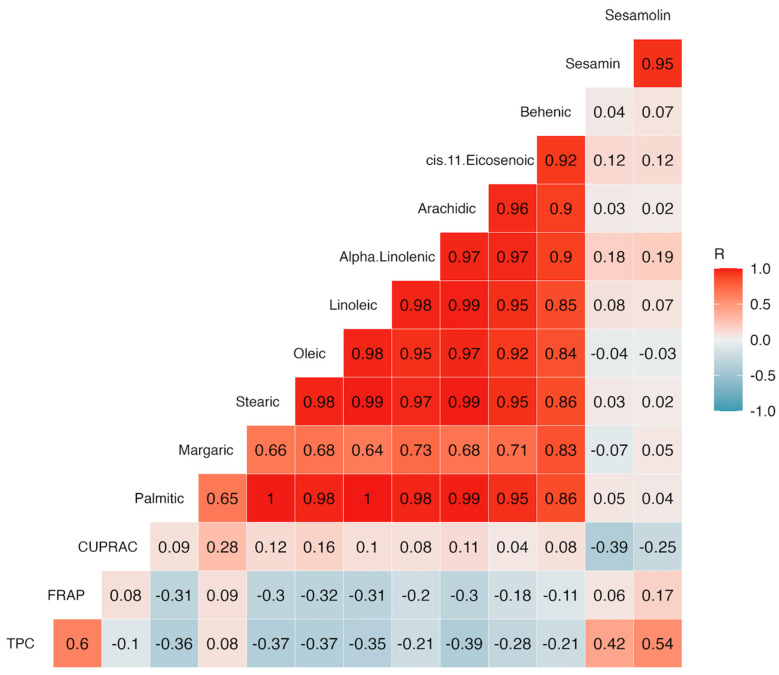
Correlogram showing the correlations amongst the phytochemical constituents of the sesame varieties studied. Correlations with R values above 0.40 or below −0.40 were statistically significant at α = 0.05.

Pearson correlation analysis was performed to explore relationships between phytochemical analytes in ground sesame extracts. A correlogram (Figure 11) was generated, where correlation coefficients were interpreted: negative values denote negative linear correlations, positive values denote positive linear correlations; 0 indicates no linear correlation, 0–0.3 signifies weak linear correlation, 0.3–0.7 indicates moderate linear correlation, and 0.7–1 indicates a strong correlation [95].

The correlation analysis revealed negative relationships between fatty acids and some antioxidant activity assays. The TPC, CUPRAC, and FRAP were not significantly correlated with any of the fatty acids, and only TPC and FRAP showed a moderate positive association with one another (r = 0.6, *p* < 0.05). (Zhou and Yu [96] found significant correlations among CUPRAC, FRAP, and total phenolics in cauliflower genotypes, with moderate positive associations between CUPRAC and FRAP (r = 0.71), CUPRAC and TPC (r = 0.470), and FRAP and TPC (0.504). These correlations were attributed to higher TPC values, possibly due to other individual compounds acting as binding agents. The varying results obtained from different techniques for assessing antioxidant activity suggest the need for further exploration using alternative analytical methods.

Again, no significant correlations were found between FRAP or CUPRAC and the major lignans (sesamin and sesamolin); however, TPC showed a moderate positive correlation with sesamin (r = 0.42, *p* < 0.05) and sesamolin (r = 0.54, *p* < 0.05). Previous studies have shown minimal antioxidative effectiveness of lignans like sesamin and sesamolin in traditional in vitro evaluations [97], but they can become potent antioxidants through in vivo metabolic alterations. Recently, Ruslan et al. [61] observed a significant negative correlation (r = −0.976, *p* < 0.01) between TPC and EC_50_ FRAP in sesame seed extracts. The authors attributed these results to increased TPC values found in sesame extracts, with higher values in dark-colored than in light-colored extracts. Moreover, a more recent report [98], claimed significant correlations between FRAP and TPC (r = 0.708, *p* < 0.01), together with other correlations between minor compounds. These findings suggest that the total phenolics may contribute minimally to the overall antioxidant activity, possibly due to synergistic interactions among various phenolic compounds and the major phenolics. Furthermore, CUPRAC and FRAP assays specifically measure unique compounds and functional groups, indicating that the antioxidants present in sesame extracts may not effectively reduce oxidants (ferric and cupric) in the present study [99]. 

Similarly, the strong correlation between sesamin and sesamolin in this study was consistent with findings from previous studies [60,100]. However, the correlation coefficient between sesamin and sesamolin in this study (r = 0.95, *p* < 0.05) was higher than those reported in studies conducted in Texas [100] and India [60], with correlation coefficients of r = 0.69 and r = 0.55 (*p* < 0.05), respectively. These differences could be attributed to genetic variations and the physical attributes of the sesame genotypes used in the respective studies. Additionally, associations between sesamin and sesamolin across different seed colors have demonstrated wider disparities, with stronger correlations observed in darker sesame genotypes (r = 0.77) compared to lighter-seed varieties (r = 0.23) [75].

On the other hand, the relationships between palmitic and linoleic acids, palmitic and arachidic acids, and stearic and arachidic acids showed significant, strong positive correlations in this study (Figure 11), which aligns with results from a previous study [86]. Voelker and Kinney [101] suggest that the synthesis of 18-carbon fatty acids involves a crucial elongation step of 16-carbon acyl chains, followed by desaturation. When this process is impaired, leading to deficiencies, the levels of 18-carbon fatty acids decrease while palmitic acid content increases in plant tissues. This deficiency likely contributes to the observed correlations. These insights indicate the potential to manipulate palmitic acid levels in sesame varieties through recurrent selection for oleic acid content. 

Contrastingly, previous findings in Indian sesame genotypes [60] reported weak negative relationships between certain fatty acids. Notably, these authors found strong negative correlation coefficients between oleic and linoleic acids (r = −0.96, *p* < 0.01), while a more moderate negative relationship was observed between palmitic and stearic acids (r = −0.44, *p* < 0.01) and palmitic and arachidic acids (r = −0.40, *p* < 0.01). All these opposing associations in Indian sesame genotypes suggest a combined influence of environmental conditions and inherent genetic characteristics [60,102] and contrasted with the strong positive correlations between these fatty acids observed in the present study. In addition to deficit irrigation stress, factors such as genetics, growing location, and environmental variations could significantly contribute to the variations in fatty acid composition in sesame seeds, as evidenced by other studies [60,100].

This study highlights intricate relationships among phytochemicals in sesame seed extracts, suggesting varying influences on total antioxidant capacity. Thus, it strongly advocates further investigation into key bioactives and alternative analytical protocols to better understand correlations between assays and antioxidant activity across different sesame genotypes [103].

## 3. Materials and Methods

### 3.1. Field Trial Information

Field trials were conducted at Alton Downs, Queensland using a two-factorial randomized complete block design (RCBD) experiment employing genotype and irrigation treatments. Eleven cultivars of black sesame were assessed for their response to available moisture (well-watered and water-deficient). Each cultivar had three field replications under well-watered conditions (100% crop evapotranspiration demand, ET_C_) and three replications under water-deficient conditions (50% ET_C_). These irrigation treatments are referred to as water-deficient (WD) for 50% ETC and well-watered (WW) for 100% ETC.

**Figure 12:**
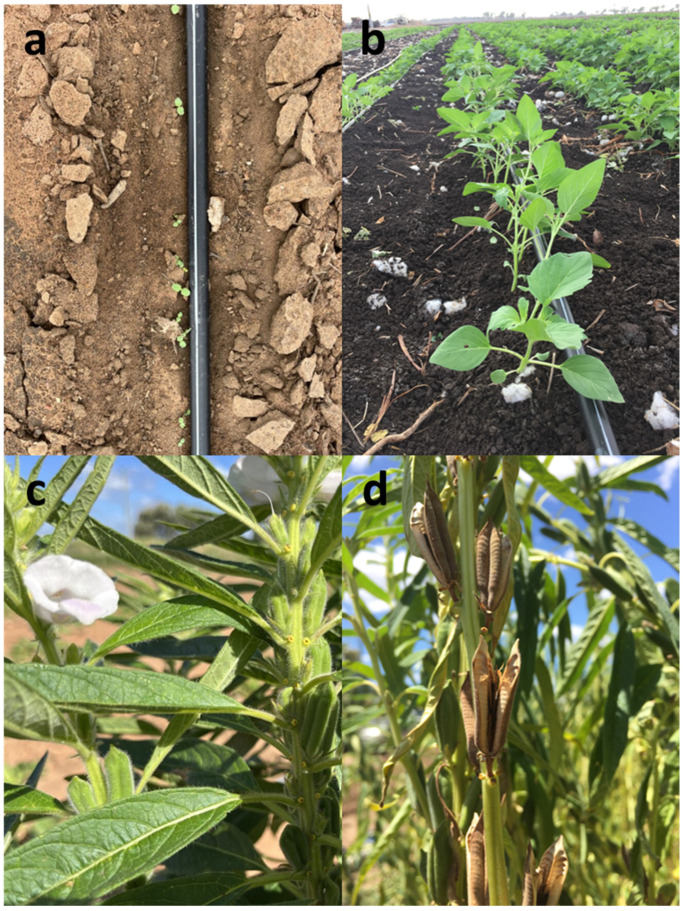
The growth of the sesame crop at different stages: (**a**) seedling; (**b**) young growth; (**c**) flowering; and (**d**) harvest.

Sowing took place in mid-November 2019, with plants maintained at 100% and 50% ET_C_ until the late bloom stage. Harvesting occurred in mid-March 2020 at Alton Downs, Queensland [104]. Photographs of the crop at different growth stages are shown in Figure 12.

After harvest, the three field replicates of each line (cultivar) were manually cleaned and air-dried until pods reached complete dryness. Threshing was the next step, using different sieve sizes [104]. Subsamples of 250 g were oven-dried (60 °C for 24 h) to a constant moisture content ranging from 2.6–5.7%.

### 3.2. Sesame Seed Material

The black sesame seed samples from the field trials were sourced from an Australian seed technology company “AgriVentis Technologies Pty Ltd.” (Sydney, Australia) (https://www.agriventistechnologies.com.au/; accessed 5 Mar 2024). Full details of these genotypes are provided in Table 1; throughout the manuscript they are referred to by their respective variety numbers (1, 2, 4, 5, 7, 8, 10, 12, 13, 17, and 18) for simplicity. It should be noted that some of these genotypes are not currently commercially grown. 

Samples (50 g) from each seed type were ground into a fine powder using an electric grinder (Breville BCG200 Coffee and Spice Grinder) following established protocols [105]. The powdered samples were then stored in sterile screw cap containers in darkness at room temperature until further analysis.

### 3.3. Reagents and Chemicals

All reagents used were of analytical grade and purchased from Chem-Supply Australia Scientific (Port Adelaide, SA, Australia). Hexane (>97% purity) was purchased from Sigma-Aldrich Australia. Anhydrous sodium sulphate was purchased from May and Baker Ltd. (Shrewsbury, Berkshire, UK). Dichloromethane (DCM) was purchased from Merck Australia (Sydney, Australia). Unless otherwise specified, all dilutions and assay preparations were performed using Milli-Q^®^ water (Merck Millipore; Bayswater, Victoria, Australia) and stored at 4 °C until required for use.

### 3.4. Sesame Standards

Individual lignan standards, including sesamin (>98% purity) and sesamolin (>98% purity) were obtained from CSA Scientific (Port Adelaide, SA, Australia). Stock standard solutions (1 mg/mL of sesamin and sesamolin) were prepared in methanol individually and then stored in the dark at 4 °C. A reference standard solution for food analysis, namely, Restek Food Industry FAME Mix (REST-35077), was obtained from Shimadzu (Rydalmere, Sydney NSA, Australia). Stock standard solution (1 mg/mL of FAME mix) was prepared in DCM.

### 3.5. Moisture Content

The moisture content of the ground sesame seeds (3 g) was determined as the mass percentage following the method described by Grabe [106]. A single sample from all three replicates of each variety and treatment were weighed onto pre-weighed watch glasses and dried in a Memmert oven (UM400 with natural convection, V: 220 V–50 Hz, Power: 1400 W, Germany) set at 120 ± 1.0 °C until a constant weight was achieved, typically for 18 min.

### 3.6. Sample Extraction

The methanolic extraction protocol previously developed by our laboratory [107] was used in this study. Approximately 2 g of ground sesame seed powder underwent duplicate extractions. Firstly, 7 mL of 90% *v*/*v* aqueous methanol was added to the powdered and mixed for 60 min on an end-over-end rotary mixer (RM4, Ratek, Australia) at 50 rpm. After centrifugation at 1000 g for 10 min (Heraeus X1 Multifuge, Thermo Fisher Scientific, Scoresby, Australia), the supernatant was collected. Another extraction was performed on the pellet with 7 mL of 90% methanol, followed by mixing for 20 min. The combined supernatants were vacuum filtered through a 0.45 μm Advantec^®^ filter paper (Advantec MFS; Dublin, CA, USA) and adjusted to 14 mL with 90% methanol. The methanolic extracts were stored at 4 °C in the dark until further analyses. Extractions and subsequent analysis of TPC, FRAP, and CUPRAC were performed in duplicate.

### 3.7. Total Phenolic Content (TPC)

Total phenolics were determined through a modified Folin–Ciocalteu method developed by Singleton and Rossi [108]. In this assay, 2 mL of a 1:10 diluted aqueous Folin–Ciocalteu (FC) reagent was combined with 400 µL of the sample extract and allowed to incubate in darkness at room temperature for 10 min. Subsequently, 2 mL of 7.5% *w*/*v* aqueous sodium carbonate solution was added, and the samples were vortexed for 30 s before being incubated in a covered water bath at 40 °C for 30 min. Absorbance readings at 760 nm were measured with a UV spectrometer (Thermo Scientific Genesys 10S UV–Vis, MA, USA), using Milli-Q^®^ water as the blank. The TPC was calculated based on the equivalent absorbance of gallic acid in the range of 20–120 mg/L (r^2^ = 0.999). Results were expressed as milligrams of gallic acid equivalents (GAE) per 100 g of dry sample weight (mg GAE/100 g DW).

### 3.8. Ferric-Reducing Antioxidant Power (FRAP)

The ferric-reducing antioxidant power (FRAP) assay, developed by Benzie and Strain [109], was conducted to assess the total antioxidant capacity of sesame seed varieties. The FRAP reagent was freshly prepared by combining 300 mM sodium acetate buffer (pH 3.56), 20 mM aqueous ferric chloride, and 10 mM 2,4,6-tripyridyl-S-triazine (TPTZ) in a ratio of 10:1:1, respectively. Pre-equilibrated (37 °C) FRAP reagent (3 mL) and 100 µL of sample extract were briefly vortexed and incubated in a covered water bath at 37 °C for 4 min. Absorbances were measured at 593 nm using a UV spectrometer (Thermo Scientific Genesys 10S UV–Vis). The FRAP values were calculated based on the equivalent absorbance of Trolox across the range of 10–175 mg/L (r^2^ = 0.999). Results were expressed as milligrams of Trolox equivalents (TE) per 100 g of dry sample weight (mg TE/100 g DW).

### 3.9. Cupric Ion-Reducing Antioxidant Capacity (CUPRAC)

The CUPRAC assay, adapted from Apak et al. [99], was performed by combining 1 mL of 10 mM aqueous copper (II) chloride, 1 mL of 1 M aqueous ammonium acetate, 1 mL of Milli-Q^®^ water, and 1 mL of freshly prepared 7.5 mM ethanolic neocuproine solution with 100 µL of the sample extract. After vortexing for 30 s, the mixture was incubated in a covered water bath at 50 °C for 30 min. Absorbances were measured at 450 nm using a UV spectrophotometer (Thermo Scientific Genesys 10S UV–Vis). The CUPRAC values were calculated based on the equivalent absorbance of Trolox (TE) in ethanol solution across the range of 50–600 mg/L (r^2^ = 0.997). Results were expressed as milligrams of Trolox equivalents (TE) per 100 g of dry sample weight (mg TE/100 g DW).

### 3.10. Determination of Lignans in Sesame Seeds Using HPLC Analysis

**Figure 13:**
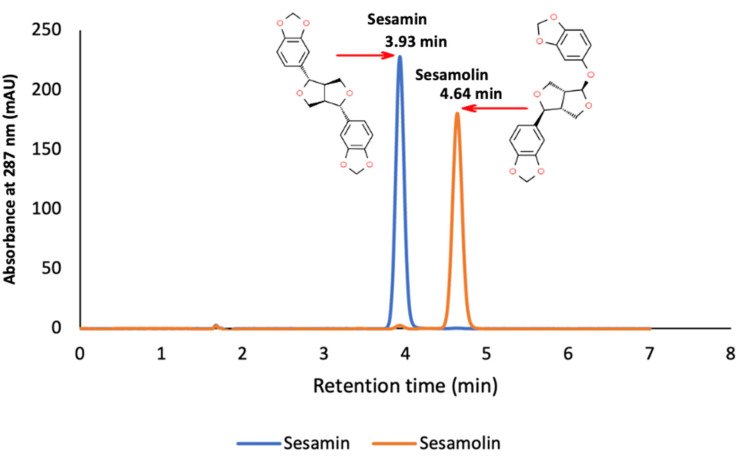
Annotated HPLC chromatogram from sesamin and sesamolin standards. (Chemical structures of sesamin and sesamolin were adapted from Sahin et al. [7]).

Sesamin and sesamolin in each methanolic sample extract (in duplicate) were quantified via HPLC analysis, following a modified protocol by Wu et al. [110]. Samples were filtered through a polytetrafluoroethylene (PTFE) membrane (Livingstone 0.45 µm) before injection into an Agilent 1100 HPLC system equipped with a G1313A autosampler, G1322A vacuum degasser, G1311A quaternary pump, and G1365B multi-wavelength detector module. An Agilent Eclipse XDB-C_18_ reversed-phase column (150 × 4.6 mm; 5 µm particle size) with a 10 µL injection volume was used. Isocratic elution with deionized water and methanol (20:80 *v*/*v*) 0.8 mL/min was applied. The total run time was 10 min, with a post-run flush time of 3 min. Retention times for sesamin and sesamolin (3.9 and 4.6 min, respectively) were verified with authentic standards (Figure 13). External standard solutions for both analytes (r^2^ = 0.999) in methanol (0–300 mg/L) were injected into the HPLC-DAD system to assess linearity between concentration, peak areas at 287 nm, and retention time values.

### 3.11. Preparation of Fatty Acid Methyl Esters (FAME) from Sesame Seed

The transesterification of sesame fatty acids followed a modified sodium methoxide protocol as reported by O’Fallon et al. [111]. For FAME synthesis, 50 mg of each powdered sesame seed (in duplicate) was mixed with 2 mL of 0.4 M sodium methoxide and incubated in a water bath at 55 °C for 1.5 h with periodic shaking every 20 min. Saturated sodium bicarbonate solution (2 mL) and 3 mL of hexane were then added to the tube and vortexed briefly. After centrifugation (Heraeus X1 Multifuge, Thermo Fisher Scientific, Melbourne, Vic, Australia) at 1000 g for 10 min, the upper hexane layer containing the FAME solution was removed using a glass Pasteur pipette into a 16 × 125 mm screw-cap Pyrex culture tube. Subsequently, 1 mL of Milli-Q^®^ water was added to the tube, and the lower aqueous layer was discarded. This process was repeated twice more. Sodium sulphate (0.5 g) was then added to each tube containing the washed FAME solution, followed by brief vortex-mixing. The FAME solution was transferred into a 2 mL plastic tube using a 3 mL disposable syringe (Livingstone–Luer slip tip), filtered through a 0.45 mm porous filter (PTFE membrane), diluted with hexane (1:9), and stored in amber GC glass vials at 4 °C until required for GC–MS analysis.

### 3.12. FAME Analysis by Gas Chromatography–Mass Spectrometry (GC–MS) of Sesame Seed Extracts

Fatty acid methyl esters were quantified using a GC–MS protocol based on previous studies [112] with slight modifications. Analysis was performed on a single quadrupole Shimadzu QP2010 Plus system equipped with an autoinjector/autosampler (AOC-20i/s) and a Restek FAMEWAX column (30 m × 0.32 mm I.D. × 0.25 µm thickness). The injection volume was 0.5 μL in split mode (split ratio = 10) at an injection temperature of 250 °C. Helium served as the carrier gas at a column flow rate of 2 mL/min. The oven temperature began at 195 °C, ramping at 5 °C/min to reach 240 °C, where it remained for 1 min. The total run time was 10 min, with the ion source and mass spectrometer interface both held at 230 °C.

For exploratory purposes, FAMEs were identified via scanning mode, with data acquisition from 50–500 *m*/*z*. Identification was based on comparison of mass spectra with the NIST14 and NIST14s libraries (https://chemdata.nist.gov/; accessed on 14 April 2023).

Consequently, FAMEs were confirmed by matching retention times with those of authentic external standards from the Restek Food Industry FAME Mix (REST-35077). For quantitative analysis, a selected ion monitoring (SIM) method was developed targeting 30 of the 37 f FAMEs in the Restek Food Industry FAME Mix.

External standard calibration was performed to quantify each individual FAME present in the sample.

### 3.13. Data Analysis

Statistical analysis was performed in R Studio, running version R-4.1.2 (R Core Team, 2021) (9). Data were presented as averages ±1 σ, with the samples categorized by variety and irrigation treatments (WD and WW) for factorial analysis. Two-way analysis of variance (ANOVA) was performed to evaluate the effects of variety and irrigation on phenolics, antioxidant activity, lignans, and fatty acids. Tukey’s adjustment was applied for comparing treatment averages, with outliers removed using Grubb’s test (*p* < 0.05). Results are reported on a dry weight (DW) basis. Pearson’s correlation coefficients were calculated to assess significant correlations (*p* < 0.05; two-tailed) amongst phenolics, antioxidant activities, lignans, and fatty acids.

## 4. Conclusions

The present study provides strong evidence that Australian-grown black sesame seeds are a promising source of bioactive compounds which can offer potential health benefits. The different analyses carried out on field-grown sesame samples indicate that specific varieties responded positively to water deficit (WD) conditions, showing increased levels of TPC, FRAP, CUPRAC, and lignans (i.e., sesamin and sesamolin). Varieties better adapted to water deficit were 1, 2, 4, 5, 7, 8, 10, and 12, which recorded the most representative TPC values. With regard to the total antioxidant activity, varieties 1, 4, 7, and 12 showed the best response to water deficit, as per their recorded increased FRAP values. Meanwhile, varieties 7, 10, 12, 13, and 18 demonstrated the highest CUPRAC values, with no significant variations between the irrigation regimes applied in field trials. Moreover, the lignan contents (sesamin and sesamolin) were greater in varieties 1, 2, 5, and 8 and responded better to the water deficit regime in these varieties. In particular, variety 10 also showed increased sesamolin content between the two irrigation conditions. Furthermore, a balanced ratio of unsaturated and saturated fatty acids (Σ UFA/Σ SFA ratio) was observed in all eleven field-grown sesame samples, with the ratios ranging from 1.86–2.34. This is well above the target value of 0.45 and is indicative of optimal health benefits. Meanwhile, the ω-6/ω-3 PUFA ratio varied from 33.7–65.5, with lower ratios in varieties 2, 4, 5, 8, and 18 under the water deficit environment. The recorded sesame traits could be potentially cultivated as the most promising lines for widespread adoption.

This study provides valuable information on the nutritional value of Australian-grown black sesame seed, which may be useful for agricultural stakeholders such as food technologists and nutritionists. It also highlights that future investigations should consider other crucial factors such as post-harvesting conditions, environmental conditions, plant architecture, and genetic factors. Conducting comprehensive investigations will help identify the most promising cultivars for long-term nutritional quality and ensure that the health benefits of Australian-grown black sesame seeds are maximized.

## Figures and Tables

**Table 1:** Details of the sesame genotypes investigated in this study. Data are sourced from [104].

No.	Genotype Code	Genotype Name	Branching Habit	Maturity	Irrigated Yield (kg/ha) ^1^	Rainfed Yield (kg/ha)	Rank (Irrigated)	Rank (Rainfed)	Difference
1	AVTBS#1	Third Gen Moriah	Multiple branching	Mid-maturing	2228	1562	10	8	2
2	AVTBS#2	Black eyes	Multiple branching	Late maturing	1275	1086	15	15	0
4	AVTBS#4	Black patch	Multiple branching	Early maturing	2449	2024	3	1	2
5	AVTBS#5	Black prince	Multiple branching	Early maturing	2276	1194	8	14	−6
7	AVTBS#7	Hammond	Multiple branching	Mid-maturing	1950	1520	13	9	4
8	AVTBS#8	Joshua IV	Multiple branching	Early maturing	2340	1565	7	7	0
10	AVTBS#10	Konji-SV	Multiple branching	Late maturing	2157	1420	11	12	−1
12	AVTBS#12	Morriah–B	Multiple branching	Early maturing	2244	1695	9	5	4
13	AVTBS#13	Black Watch * A.T. Moriah	Multiple branching	Mid-maturing	2348	1913	6	3	3
17	AVTBS#17	NA/1	Multiple branching	Mid-maturing	2549	1738	1	4	−3
18	AVTBS#18	A.T. Moriarty	Multiple branching	Early maturing	2415	1955	4	2	2

^1^ Yields are hand-harvested yields.

## Data Availability

The data supporting the findings of this study are available upon request from the corresponding author.
